# Evaluation of a charity-led secondary breast cancer support day: a model of survivorship care

**DOI:** 10.3332/ecancer.2019.991

**Published:** 2019-12-12

**Authors:** Barbara S Baker, Jean Marshall, Caroline J Hoffman

**Affiliations:** 1 Breast Cancer Haven, Dowgate Hill House, 14-16 Dowgate Hill, London EC4R 2SU, UK; 2 Breast Cancer Haven, 4-5 The Gateway West, East Street, Leeds LS9 8DA, UK

**Keywords:** secondary breast cancer, support, survivorship, model of care

## Abstract

It has been estimated that there are 36,000 women living with secondary breast cancer in the UK. Many feel isolated and unsupported, their information needs unmet and unaware of available support services such as palliative care or support groups that could help to improve their quality of life.

To try to address these needs, a monthly support day was established in 2014 by the national UK breast cancer charity Breast Cancer Haven (BCH) at their Yorkshire centre. The support day provides an opportunity for personal introductions and discussion with other people with secondary breast cancer, to obtain information about breast cancer-related topics of their choice, to eat a healthy lunch and to experience a visualisation/relaxation session. To evaluate how helpful this support day was to its participants, they were asked to complete a feedback form at the end of the day. A total of 171 forms were completed from 26 support days during the period February 2014–July 2018.

Participants stated that they found the support day helpful, relaxing, informative, supportive and enjoyable. All except two felt it met their needs and expectations and the majority found the length of the day just right. The personal introduction and discussion session were most frequently cited as the most useful part of the day, with the majority of participants (*N* = 144, 96.7%) rating it as very or moderately helpful.

These findings show that the BCH support day, developed to address the needs of people with secondary breast cancer, is a model of survivorship care that can have a positive impact on their lives.

## Introduction

Secondary breast cancer (also known as metastatic or stage four) occurs when breast cancer cells spread to other parts of the body such as the bone, brain, liver or lungs. It is not curable, but treatment can prolong survival. Median survival is around 3–4 years [1], but, depending upon site and response to treatment, some people live for many years and others just a few months after diagnosis.

It has been estimated that there are 36,000 women living with secondary breast cancer in the UK [2], and each year, around 11,400 women die from the disease [3]. However, as a result of inadequate data collection by many NHS Trusts, there is no accurate, up-to-date figure of the number of people diagnosed with secondary breast cancer in the UK [4]. Furthermore, people living with the disease feel the care they receive is inferior to that of people with primary breast cancer, and many feel isolated, unsupported and uncared for as a result [5]. In addition, they report that their information needs are not being met and that they are not being consistently signposted to available services such as palliative care or support groups that could improve their quality of life [5].

Breast Cancer Haven (BCH) is a national UK breast cancer charity supporting people through the physical and emotional experience of primary and secondary breast cancer. BCH provides free in-depth personalised programmes of psychological support, help with treatment side effects and supported self-management activities in five community-based centres located in London, Hereford, Yorkshire, Wessex and the West Midlands, two hospital breast units in London and Worcester, plus support days across the country. The charity has helped people live well with and beyond breast cancer since February 2000.

The BCH support programme, based on a model of integrated cancer care, has been developed over 20 years by the charity’s experienced clinical team. It incorporates different forms of emotional and psycho-educational support as well as the use of complementary therapies to support physical symptoms. Regular service evaluations have been carried out to monitor its effectiveness [6, 7]. The programme consists of an initial 1-hour consultation with an experienced BCH healthcare professional to assess each person’s needs and to mutually agree on an appropriate therapy plan, followed by up to 10 hours of free individual one-to-one therapies. After 6 hours of therapy, each person’s treatment plan is reviewed with a BCH healthcare professional to monitor the effectiveness of the therapies.

Having received support from the charity after a primary diagnosis of breast cancer, people who later develop secondary tumours are offered a second support programme of therapies to help them with their symptoms and treatment side effects. In addition to their personalised support programme, a monthly support day tailored to address their specific needs has been established and evaluated at the BCH centre in Leeds, Yorkshire, since 2014. The support day provides an opportunity for personal introductions and discussion with other people in a similar situation, to obtain information about breast cancer-related topics of their choice, to eat a healthy lunch and to experience a visualisation/relaxation/meditation session. To evaluate how helpful this support day was to its participants, they were asked to complete a feedback form at the end of the day, the findings of which are reported here.

## Methods

A service evaluation was carried out on a sample of people with secondary breast cancer who had attended at least one of the monthly support days developed specifically for them at the BCH, Yorkshire centre. All people receiving help from BCH have given their consent, on registration, to be contacted for feedback about the charity’s treatment programmes.

### Participants

Participants were people with secondary breast cancer from Leeds, Halifax, Huddersfield, York, Wakefield and Bradford who had attended the BCH, Yorkshire centre in Leeds. Their ages ranged from forties to late seventies with varying levels of mobility and general health.

An email or letter was sent to all the people with secondary breast cancer on the centre’s visitor list (approximately 60) 2 weeks before each support day inviting them to attend. The numbers attending each support day averaged around 10–15.

### Secondary breast cancer support day programme

The secondary breast cancer support days are organised from 11 am to 3 pm, including a healthy lunch dividing the day into morning and afternoon sessions. Participants can attend as much or as little of the day as they like, which may be determined by, for example, their level of fatigue.

## Morning session

### Personal introductions and discussion

This 1-hour session is facilitated by an experienced therapist and group facilitator who ensures that everyone has a chance to speak if they want to and that the group feel that they are driving the content of the discussion. The aim is to provide a safe environment in which group members feel able to express themselves freely and easily while receiving support from the therapist and other group members. New members are invited to introduce themselves to the group, if they wish, by saying as little or as much as they want to about themselves.

One of the main issues that are addressed within this session is the death of any of the members since the previous meeting. This involves, first, acknowledging when someone has died and allowing a short period of silence to think about the person and their family. Group members are then invited to speak about what the deceased person meant to them and to say goodbye, if they wish to do so. Group members who were friends of the person may also have attended the funeral and want to share that experience with the group. There is also a ‘memory book’ for people to express their thoughts in writing. If someone is attending for the first time in this situation, the therapist explains the aforementioned process to them so they know what to expect and can choose to join the group after the session if they so wish.

The members are encouraged to feedback to the therapist their views on how this sensitive issue is dealt with within the group to ensure that it provides the support they need.

Other topics addressed during this session include the following.

- How people are feeling physically and emotionally.
- An update on the current situation of people who have not been able to attend for a while due to health, treatment, transport, work or other reasons.
- Treatments and their responses to them.
- Clinical trials that members are taking part in.
- When scans are due/results pending.
- Satisfaction/dissatisfaction with aspects of care or treatment—comparing different NHS Trusts.
- General lack of support for people with secondary breast cancer.
- Appreciation of support from BCH and Breast Cancer Care, and other cancer charities.

### Talks by internal/external speakers

An opportunity to experience different therapies such as Emotional Freedom Techniques, Breathing and Movement, Relaxation/Meditation during the day was available until February 2015.

This has been replaced by 1-hour talks by internal and external speakers, including breast surgeons, oncologists, psycho-oncologists and therapists, on a variety of topics, such as nutrition, pain management, fatigue, cancer treatment and mindfulness, chosen by participants.

## Healthy lunch

In between the morning and afternoon sessions, a healthy vegan lunch is provided by volunteers at BCH to cater for all tastes, with dairy and wheat as optional extras. The content of the lunch is consistent with ‘The Haven’s Guide to Healthy Eating’, developed by the charity based upon up-to-date high-quality research evidence [8, 9].

## Afternoon session

### A visualisation/relaxation session

This 45-minute session, facilitated by the same therapist as the discussion session, is conducted in a large meeting room with reclining chairs, footrests and blankets to ensure that everyone taking part feels relaxed and comfortable.

The session, with quiet background music, starts with calm breathing, followed by a short full-body relaxation and a visualisation read by the therapist. The visualisation can be based on a variety of scenarios such as a country walk, a relaxing, floating experience, healing light, a mindfulness practice and walk on a beach or through forest. In the end, there is a short time for people to relax for a while listening to the music before they are invited to have a stretch and open their eyes when they are ready.

### Evaluation of secondary breast cancer support day

Women with secondary breast cancer who attended monthly secondary breast cancer support days during the period February 2014–July 2018 were asked, at the end of the day, to complete a feedback form developed by BCH therapists to evaluate the helpfulness and suitability of the support day’s programme. They were asked to rate the talks and sessions (very helpful, moderately helpful or not helpful), whether the day met their needs and expectations (yes/no) and, if no, to indicate what was missing, how they found the length of the day (too long, about right, too short), and to comment on which part(s) of the day they found most useful.

A total of 171 feedback forms were collected from 26 support days (completed feedback forms were not available for all support days conducted during this period).

### Data analysis

Quantitative data from completed feedback forms were analysed using descriptive statistical analysis, giving a frequency analysis of responses (numbers and percentages) to each question.

Free text responses about the most useful part(s) of the support day were analysed by thematic analysis of the named sessions, accompanied by frequency analysis of the themes identified.

## Results

### Personal introductions and discussion

The majority of the 149 participants who attended/responded rated the session as very helpful (*N* = 125; 83.9%), with a further 19 (12.8%) rating it as moderately helpful. Only five people (3.3%) did not find the session helpful.

One participant commented that ‘*Meeting others and hearing their stories makes you feel not so alone’* (P1).

### Talks by internal/external speakers

**Figure 1.**
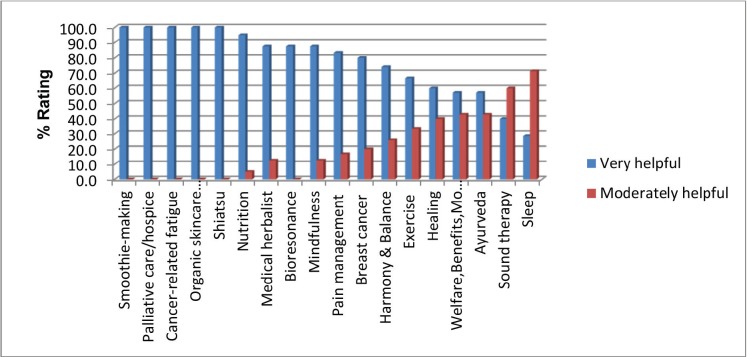
Percentage rating of talks by internal/external speakers.

The majority of the talks by internal/external speakers, which covered a wide range of different topics, were rated as being very helpful by the majority of participants, with the exception of the sleep and sound therapy sessions which were mostly reported as being moderately helpful by a small number of participants. The welfare, benefits and money advice and Ayurveda sessions had almost equal numbers of participants who rated them as very or moderately helpful. However, the numbers who attended were small (*n* = 7). With the exception of one person who attended the bioresonance talk, none of the participants found the talks by external speakers unhelpful (Table 1, Figure 1).

One participant made the following comment ‘*Masses of information I never knew. Made a visit to the hospice far less daunting’* (P2).

### Visualisation/relaxation session

Nearly all the participants (*N* = 102; 91.9%) who attended/responded found the afternoon session of visualisation/relaxation very helpful, whilst nine people (8.1%) felt it was moderately helpful. None of the participants found the session unhelpful. As one participant stated *‘I could do with an extra 15 mins on this session as it’s so relaxing and helpful’* (P3).

### Support day meets expectations and needs?

All participants, except two, who answered the yes/no question (*N* = 159; 98.8%) agreed that the support day met their needs and expectations. One participant commented ‘*Yes—I was made very welcome. The structure of the session and topics were interesting’* (P4).

### Length of the support day

The majority of participants (*N* = 153; 94.4%) thought that the length of the day was about right (Table 2).

### Most useful part(s) of the support day

There were 204 responses to the question about the most useful part(s) of the day. (Some respondents gave more than one answer.)

The part of the day most frequently found to be useful by respondents was the opportunity to introduce themselves and talk and share information with others in a similar situation (*N* = 72; 35.3%), followed by talks by internal/external speakers (*N* = 48; 23.5%) and the visualisation/relaxation sessions (*N* = 43; 21.1%). There were only three negative comments: the talks could have been longer, lunchtime felt too long, and there was too much free time (Table 3).

### Any other comments to help improve the support day

The most frequent responses were positive comments about the support day (well run, enjoyable, helpful, relaxing, informative, comforting, supportive, welcoming, invaluable day, very good structure, look forward to, feel better after) and appreciation of the support received (*N* = 58/87; 66.7%).

A longer Personal Introductions and Discussion session allowing more interaction with others was requested by six participants, and three others suggested that name labels would be helpful (this suggestion has now been acted upon and first name badges are provided). Other individual suggestions were made by 14 participants including help with lymphoedema, a discussion on how to honour a lost member, armchair exercise and help with dating for cancer patients.

There were six negative comments: four people complained about a distracting noise outside the room during relaxation, one person felt that discussions were dominated by individuals and another found that the bioresonance session was unhelpful.

## Discussion

People with secondary breast cancer say that often they are not getting the care and support they need to live well nor are their information needs being met [5]. The BCH support day at its centre in Yorkshire was specifically developed to provide a safe, welcoming environment in which emotional/psychological support is combined with an information component delivered by internal/external speakers to try and address these unmet needs.

Participants of the monthly support day for people with secondary breast cancer found it helpful, relaxing, informative, supportive and enjoyable. All except two felt it met their needs and expectations and the majority found the length of the day just right. They most frequently rated the time to talk to each other about themselves and their issues as the most useful part of the day, with nearly three-quarters of them rating it as very helpful.

New treatments for secondary breast cancer are prolonging the length of life for many [10], but their needs and concerns, particularly the long-term, life-altering impacts of the disease and its treatment, are poorly understood. The most prevalent, persistent and disabling symptoms they experience include depression, anxiety, sleep disturbance, pain and fatigue [11–14].

Improvement of some psychological symptoms has been reported for group psychological interventions such as mindfulness-based stress reduction, cognitive behavioural therapy and supportive-expressive therapy [15, 16]. The latter two therapies also appear to be effective in improving survival at 12 months [16]. The Personal Introductions and Discussion session offered as part of the BCH support day is based on a similar approach to that of supportive-expressive group therapy. The latter focuses, via a facilitator, on fostering mutual support and helping people face and deal with their disease-related stress, and has been shown to help reduce distress in people with secondary breast cancer [17].

Participants of the support day particularly valued the discussion session, more frequently rating it as the most useful part of the day. This concurs with the findings of an evaluation of information and support interventions developed for patients with secondary breast cancer at the Christie Hospital in Manchester, which showed that meeting others in similar situations was the most frequently reported reason for attending the events [18]. Furthermore, some participants took the opportunity before the first session started and during the lunch break, to talk with others in the group unfacilitated and to keep in contact with each other via texts, phone calls and as part of a WhatsApp group, demonstrating the need for peer as well as professional support. Such interactions helped to reinforce the feeling that they were not alone in dealing with their diagnosis. As one participant commented ‘*A lovely positive group—felt very supported by the other women*’ (P5).

Dealing with the issue of the death of a member of the group was an important topic of discussion: feedback to the therapist about the way this was handled within the group discussion showed that it was addressed in a way that the majority of the group found appropriate. One participant commented ‘*Memory book great idea. I bought a candle for (name) to light each month until it is no more*’ (P6). A reduction in traumatic stress symptoms associated with such issues has also been reported using supportive-expressive group therapy in women with secondary breast cancer [17].

Findings from large international surveys of women with secondary breast cancer have revealed their expressed need for good, accurate, in-depth information about their disease, standard and emerging treatments, and ways of coping with side effects and symptoms [19, 20]. This was reflected in the wide range of topics for talks by internal/external speakers chosen by the participants of the support day. The participants frequently found this informational part of the day very helpful, suggesting that at least some of their informational needs were being met. As stated by one participant ‘*Very interesting and informative. Makes it easier to understand the different treatments and why NHS/NICE/ etc restrictions*’ (P7).

The majority of the participants found the relaxation with visualisation (guided imagery) afternoon session very helpful. Relaxation and guided imagery, alone or in combination, have been demonstrated to be effective interventions to decrease the psychological impact (mood, anxiety and depression) as well as alleviate the adverse effects (nausea and vomiting) in breast cancer patients undergoing chemotherapy treatment [21–23]. Several of the participants commented on how much they enjoyed the relaxation session. One commented that ‘*The relaxation at the end is just perfect*’ (P8).

As far as we are aware, this is the first reported evaluation of a model of survivorship care developed specifically for people with secondary breast cancer. This approach would also be applicable and potentially beneficial for any breast cancer survivors as it is similar in content to other positively evaluated group support events offered at BCH [6, 7]. Further research is needed in a larger sample of patients to explore and identify those components that address their unmet needs most effectively.

## Conclusions

Many people living with secondary breast cancer feel isolated and in need of specific support to improve their quality of life and to help with a variety of symptoms and side effects. BCH’s monthly support days offer a safe, welcoming environment in which psychoeducational and social support tailored to address these unmet needs is provided. Evaluation of this model of survivorship care shows that such an approach can have a positive impact on people living with secondary breast cancer.

## Conflicts of interest

Dr Barbara Baker and Dr Caroline Hoffman are employees of BCH. Jean Marshall is a self-employed therapist and group facilitator who runs the BCH secondary breast cancer support days.

## Funding

No external funding was received for this evaluation.

## Figures and Tables

**Table 1.** Rating the helpfulness of talks by internal/external speakers.

Subject of talk	Very helpful	Moderately helpful	Not helpful	Didn’t attend/ respond	Total
Smoothie-making	16				16
Palliative care and role of hospice	8				8
Cancer-related fatigue	7				7
Organic skincare products	5				5
Shiatsu	3				3
Nutrition	19	1			20
Medical herbalist	7	1			8
Pain management	5	1			6
Breast cancer	4	1			5
Bioresonance	7		1	1	9
Exercise	4	2			6
Reiki (healing)	3	2			5
Harmony and balance	20	7		9	36
Mindfulness	14	2		10	26
Sleep	2	5			7
Sound therapy	2	3			5
Welfare, benefits and money advice	4	3			7
Ayurveda	4	3			7
Total	134	31	1	20	186

**Table 2.** Length of the day.

Response	Number (%)
Too long	4 (2.5)
About right	153 (94.4)
Too short	5 (3.1)
Total	162

**Table 3.** Comments about the most useful part(s) of the day.

Topics	Number of responses	Participant quotes
Introductions/group sharing session	72	*‘Meeting others in the same situation as myself’.* *‘Listening to other ladies about their cancer and treatment and their feelings’.* *‘An authentic discussion on loss of group members’.*
Information provided by internal/external speakers	48	*‘Discussing and learning about new treatments and what trials are on’.* Learnt that*… ‘Healthy food can be simple and easy to prepare—and tasty!’* *‘Good to know about benefits of exercise’.* *‘The* (welfare and benefits) *information was invaluable. I can claim for things that I had no idea about’.*
Visualisation/relaxation/meditation	43	*‘Relaxation is the best way to finish—leave floating’.*
Morning, afternoon or all day helpful	26	*‘I enjoyed the whole day and the different experiences. The day has given me a lift and I have had a very positive experience from the day’.*
Therapies (offered up to February 2015)	9	*‘The series on movement/breathing was excellent’.*
Lunch	4	*The length for lunch is just right*
Environment	2	‘*Lovely space for us to meet*’.
Total	204	
